# On the Therapeutic Uses of Peroxide of Hydrogen

**Published:** 1890-07

**Authors:** 


### ON THE THERAPEUTIC USES OF PEROXIDE OF HYDROGEN.

Dr. Mikhail P. Manassein, of St. Petersburg, (*Novosti Terapii*, No. 5, 1890, p. 75), emphatically draws attention to the following points in connection with the subject: 1 deg. Peroxide of hydrogen is an excellent antiseptic and disinfectant agent which deserves the most extensive use. 2 deg. The remedy proves especially valuable in cases of herpes progenitalis, soft chancres and gonorrhœa. The latter may be cured by the peroxide (in the shape of injections) in from eight to twenty-one days; soft chancres in from five to fourteen (the drug being applied in the form of lotions). 3 deg. The drug is entirely free from any odor, does not soil the patient's linen, and does not give rise to any local pain or irritation or any unpleasant general effects (even when used in strong solution). 4 deg. The peroxide affords a most reliable means for preventing any venereal infection (by men it should be injected into the urethra and used as a wash after each suspicious coition; women should inject it into the vagina and wash out their external genitals with it, both before and after each sexual intercourse). 5 deg. The opinion that the peroxide is a very unstable preparation proved to be quite erroneous; in reality, when kept in some dark and cool place the solution remains unchanged for a very long stretch of time.—*St Louis Med. and Surg. Jour.*

BOB—"My dad's a squire and gets his name in the paper every day."

TOM (contemptuously)—"That's nuthin.' My dad took Jink's liver pills and got his pictur in the paper."—*Pittsburgh Bulletin.*

